# MOVE: weight management program across the veterans health administration: patient- and facility-level predictors of utilization

**DOI:** 10.1186/1472-6963-13-511

**Published:** 2013-12-10

**Authors:** Aaron C Del Re, Matthew L Maciejewski, Alex HS Harris

**Affiliations:** 1 Center of Innovation to Implementation, VA Palo Alto Health Care System & Stanford Medical School, 795 Willow Rd, Menlo Park, CA 94025, USA; 2 Center for Health Services Research in Primary Care, Durham VA Medical Center, 411W Chapel Hill St, Ste 600, Durham NC 27705, UK; 3 Division of General Internal Medicine, Department of Medicine, Duke University, 795 Willow Rd, Menlo Park, CA 94025, USA

**Keywords:** Obesity, Treatment utilization, MOVE!, Obesity management program, Veterans

## Abstract

**Background:**

Health care systems initiating major behavioral health programs often face challenges with variable implementation and uneven patient engagement. One large health care system, Veterans Health Administration (VHA), recently initiated the MOVE!® Weight Management Program, but it is unclear if veterans most in need of MOVE!® services are accessing them. The purpose of this study was to examine patient and facility factors associated with MOVE!® utilization (defined as 1 or more visits) across all VHA facilities.

**Methods:**

Using national administrative data in a retrospective cohort study of eligible overweight (25 < = body mass index (BMI) < 30 and at least one obesity associated comorbidity) and obese (BMI > =30) VHA outpatients, we examined variation in and predictors of MOVE!® utilization in fiscal year (FY) 2010 using generalized linear mixed models.

**Results:**

4.39% (n = 90,230) of all eligible overweight and obese patients using VHA services utilized MOVE!® services at least once in FY 2010. Facility-level MOVE! Utilization rates ranged from 0.05% to 16%. Veterans were more likely to have at least one MOVE!® visit if they had a higher BMI, were female, unmarried, younger, a minority, or had a psychiatric or obesity-related comorbidity.

**Conclusions:**

Although substantial variation exists across VHA facilities in MOVE!® utilization rates, Veterans most in need of obesity management services were more likely to access MOVE!®, although at a low level. However, there may still be many Veterans who might benefit but are not accessing these services. More research is needed to examine the barriers and facilitators of MOVE!® utilization, particularly in facilities with unusually high and low reach.

## Background

### MOVE!® weight management program utilization across VA facility

The prevalence of obesity (Body Mass Index (BMI) > 30) has grown steadily over the past several decades to nearly 34% of the United States population in 2008 [1]. Obesity increases risk of developing several chronic health conditions, including cardiovascular disease, diabetes, sleep apnea and other medical conditions [2]. Further, obesity is linked to reduced quality of life, survival [1,3] and increased healthcare costs [4-6]. The prevalence of obesity among the 5.5 million patients treated yearly in the Veterans Health Administration (VHA) is similar (35%) to that of the general U.S. population [2,3,7]. To address the obesity epidemic in VHA, MOVE!® was developed in 2006 to provide weight loss programs throughout the VHA health system, based on evidence-based principles and to provide a multifaceted approach to treating and managing obesity [8-10]. Veterans are eligible for MOVE!® if they are obese (BMI ≥30) or overweight (25 ≤ BMI <30) with obesity-related conditions, younger than age 70, and have no contraindication to weight loss.

In 2010, just over 30% of obese VHA patients were estimated to have received one of the numerous obesity management interventions available in VHA (e.g., education, nutrition counseling, medication) [11] but only 2% of the total VHA outpatient population (> 5.5 million) had contact with MOVE!® [12]. It is unclear to what extent MOVE!® is reaching veterans with higher BMIs or veterans with obesity-related comorbidities who are at increased risk for adverse events.

Previous research has found that utilization of VHA obesity management services in 2002 to 2006 (prior to MOVE!®) was more likely among racial/ethnic minorities, women, younger veterans, and unmarried veterans [11,13]. Veterans with psychiatric conditions were also found to use obesity management services at higher rates if they had obesity-related comorbidities (e.g., diabetes) or filled obesogenic psychiatric medications in VHA [13].

The current analysis extends these prior studies by examining use of MOVE!® services in a national population of veterans in 2010. The purpose of this study was to describe the facility-level variability in the utilization of MOVE!® (defined as 1 or more visits) and to examine patient- and facility-level correlates of MOVE!® use, which can inform MOVE!®-related quality improvement efforts.

## Methods

### Study design and sample

**Figure 1:**
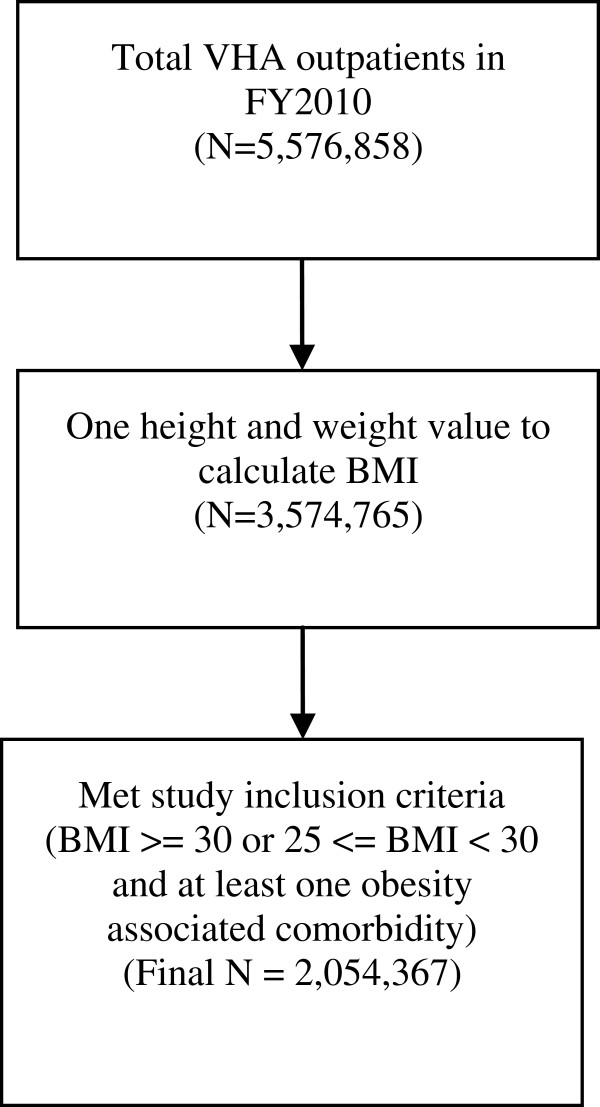
**Flowchart.** Legend: Flowchart of reduction in sample at each data step.

Using the outpatient VHA Decision Support System (DSS) database, we conducted a retrospective cohort study of all veterans from 140 Veterans Affairs Medical Centers (VAMCs) that offered the MOVE!® program in 2010 that had at least one available height and weight to calculate Body Mass Index (BMI). FY2010 was the most recent data available at the time of analysis. There is no reason to believe that this particular year is any different in promotion to clients or providers (or otherwise) than previous or later years. Of the 5,576,858 total VHA outpatients seen in fiscal year (FY) 2010 in these 140 facilities, 64% (N = 3,574,765) had at least one height and weight available to calculate BMI (Figure 1). If there were multiple values per patient available, the median value was used, among biologically plausible (i.e., height < 84 inches and weight < 700 pounds) values, to calculate BMI. Patients were retained in the final sample (N=2,054,367) if they had a BMI > = 30 or 25 < = BMI < 30 and at least one obesity associated comorbidity (e.g., diabetes, hypertension, hyperlipdemia, heart disease, congestive heart failure, cholelithiasis, osteoarthritis, low back pain, gastroesophageal reflux disease, and obstructive sleep apnea). Note that patients >70 years of age are not a targeted group for MOVE!® but we chose to analyze the total population of obese veterans in 2010 to have a more complete understanding of MOVE!® use among *all* obese veterans and for greater generalizability.

### Outcome and explanatory variables

The primary outcome of this study was MOVE!® utilization, defined as having at least one MOVE!® outpatient visit in VHA National Patient Care Databases (NPCD) in FY2010 identified using VHA clinic stop codes 372 or 373.VHA stop codes are included in all outpatient records to signify the type of clinic or treating specialty.

Several patient characteristics were obtained from the NPCD including age (categorized as age <40, 40–49, 50–59, 60–69, 70–79, 80+), gender, marital status (married or other), minority status (minority or non-minority status), BMI (defined as [703 × weight in pounds]/ [height in inches squared]) categorized into 4 levels (overweight = BMI <30, Class I Obesity = 30–34 BMI, Class II Obesity = 35–40 BMI, and Class III Obesity >40 BMI), home instability ("HOMLESS" in NPCD or clinic stop code signifying homeless or housing services or ICD-9-CM V60 indicating lack of housing), outpatient copayment status (co-pay required vs. not), geographic location, obesity diagnosis (ICD-9-CM 278.00, 278.01, 259.9, V778), obesity-related comorbidities, obeseogenic psychiatric drug prescription, and psychiatric diagnoses.

Copayment status was determined from VHA priority status, which consists of nine disability and income related categories. Although MOVE!® is a free service to all veterans requiring no co-pay, we chose to include this variable, as it correlates with both illness severity and socioeconomic status [13,14]. Geographic location was classified into urban, rural, or highly rural location, based on patients’ zip code following methods described by Kaboli & Glasgow [15]. Obesity-related comorbidities included diabetes (250, 357.2, 362.0, 366.41), hypertension (401–405), hyperlipdemia (272), heart disease (429.1, 429.0, 429.2, 429.9, 410, 411,412, 413, 414, 440), congestive heart failure (402, 404.0, 414.19, 425.4, 428, 429.1, 429.4, 997.1), cholelithiasis (574), osteoarthritis (715), low back pain (722, 724, 846, 847), gastroesophageal reflux disease (530.11, 530.81, 530.2,787.1), and obstructive sleep apnea (780.57, 786.03, 327.2, 327.20, 327.21, 327.23, 327.29). Obeseogenic psychiatric drug prescriptions included amitriptyline, clomipramine, desipramine, doxepin, imipramine, mirtazapine, nortriptyline, paroxetine, clozapine, olanzapine, quetiapine, risperidone, thioridazine, lithium, valproic acid, gabapentin, and nefazodone. Psychiatric diagnoses included every major disorder (ICD-9 codes: 294, 295, 296,297.0, 297.1, 298, 300, 301, 306, 307, 309.1, 309.8, 309.9, 310, 311, 313.1, 314.0). See Table 1 for details of the included variables.

**Table 1:** Obesity- and psychiatric-related diagnoses

**Condition**	***ICD-9-CM *** **diagnosis code**
Obesity	278.00, 278.01, 259.9, V778
Diabetes	250, 357.2, 362.0, 366.41
Hypertension	401, 402, 403, 404, 405
Hyperlipdemia	272
Coronary Heart Disease/Ischemic Heart Disease (includes CAD, MI)	429.1, 429.0, 429.2, 429.9, 410, 411, 412, 413, 414, 440
Congestive heart failure	402, 404.0, 414.19, 425.4, 428, 429.1, 429.4, 997.1
Cholelithiasis	574
Osteoarthritis	715
Low back pain	722, 724, 846, 847
Gastroesophageal reflux disease	530.11, 530.81, 530.2,787.1
Obstructive sleep apnea	780.57, 786.03, 327.2, 327.20, 327.21, 327.23, 327.29
Psychiatric Dx (any)	294, 295, 296,297.0, 297.1, 298, 300, 301, 306, 307, 309.1, 309.8, 309.9, 310, 311, 313.1, 314.0

In addition, we calculated engagement in "other obesity-related care" (Current Procedural Terminology codes S9449, S9451, S9452, S9470, G0270, G0271, 97110, 97113, 97530, 97150, 97802, 97802, 97803, 97804; outpatient clinic codes 123, 124, 139, 140, 708, 709; weight loss medications orlistat, phentermine and sibutramine; ICD-9-CM codes 43.89, 44.31, 44.38, 44.39, 44.68, 44.69, 44.95, 44.96, 44.97, 44.98, 45.51, 45.91) which involved any other obesity care, such as nutritional consultation or bariatric surgery. However, we found that among patients receiving MOVE!, 87.03% had received other obesity care. Because other obesity care is essentially a proxy for our outcome, including it as a predictor substantially changes the interpretation of the other predictors. Given that our aim was to predict MOVE! utilization, not MOVE! utilization conditional on having received other obesity care, we did not include this term in our models at the patient-level.

Facility -level predictors were derived by aggregating patient characteristics by facility (N = 140) and enabled examination of the relationship between overall facility characteristics and MOVE!® utilization. The following facility characteristics were calculated, based on percentage of patients in the facility meeting criteria: facility rates of male gender, minority status, home instability, outpatient copay, rural geographic residence, BMI, obesity-related comorbidity, obeseogenic psychiatric drug prescription, psychiatric diagnosis, and provisions of other obesity care.

### Analytic plan

To describe and explore variability in facility-level rates of MOVE!® utilization, we calculated the rate of MOVE!® receipt (number of obese patients who had at least one MOVE!® visit divided by the total number of obese patients in the facility) in each of the 140 VHA facilities. Then, we examined patient factors associated with the binary patient-level response variable (MOVE!® utilization: 0 = no, 1 = yes), which was estimated using generalized linear mixed effect models with a random effect for facility to account for the clustering of patients within VHA facilities. Non-significant variables were trimmed from these models, resulting in the final multivariate model. The mixed-effect regression models were conducted using the GLIMMIX function within the SAS statistical software (version 9.2) and additional analyses, including graphics with the ggplot2 package and advanced mixed-effects modeling procedures with the lme4 package were conducted within the R statistical software (version 3.0.1). The VA Palo Alto Health Care System’s research office and Stanford University’s Human Research Protection Program approved this project.

## Results

**Figure 2:**
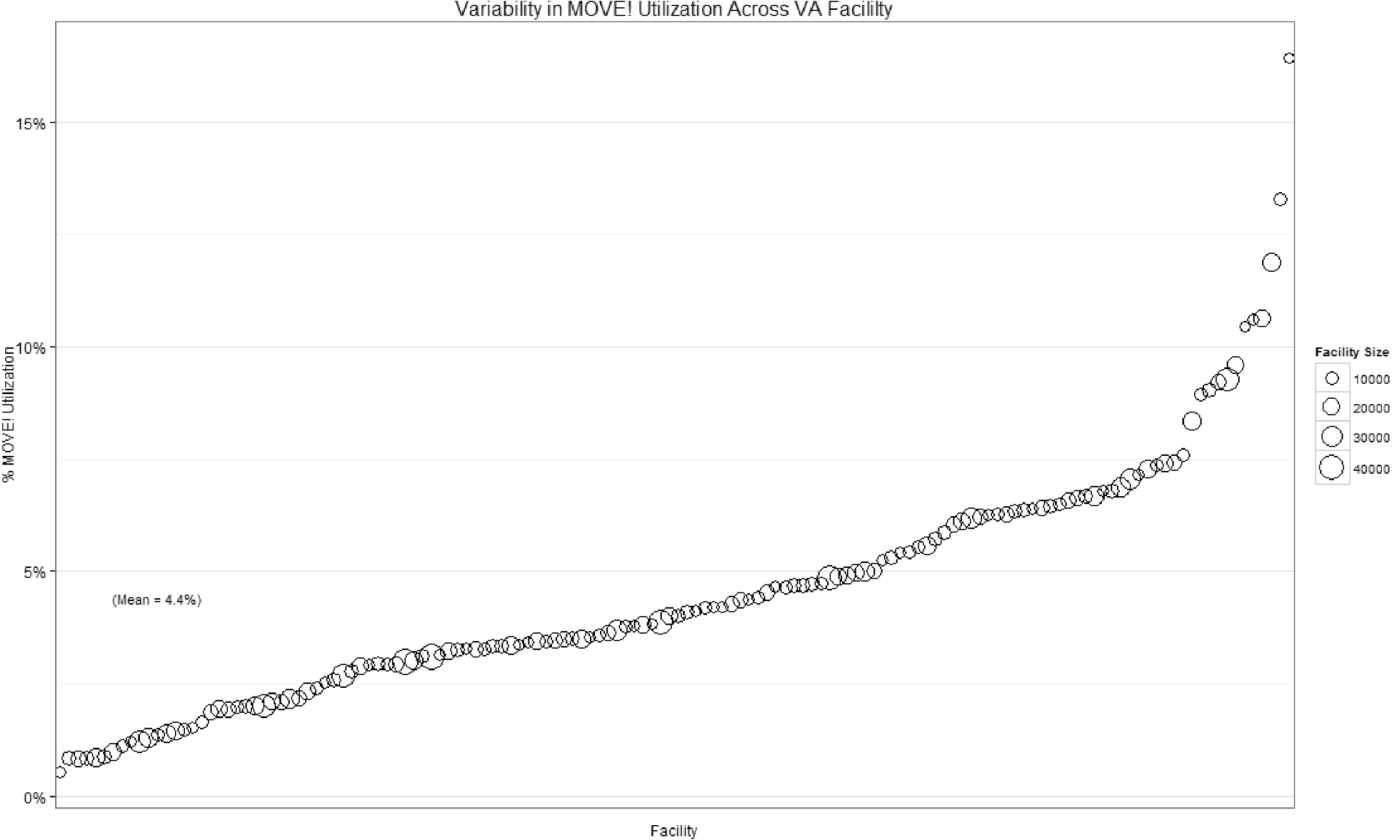
**Variability in MOVE! utilization across VA facility.** Legend: Displayed is the variability in facility level percentage of patients utilizing MOVE services. The 140 VA facilities are ordered from smallest to largest percentage and the size of each point represents the number of patients within each respective facility.

In FY2010, 4.4% (*n* = 90,238) of all veterans in the final analytic sample (N=2,054,367) had at least one MOVE!® visit, with an average of 4.9 (*SD* = 8.4, median = 2.0) MOVE!® visits in 2010 among those with at least one visit. Within the 140 VHA facilities, the proportion of patients who received MOVE!® treatment ranged from 0.05% to 16% (mean = 4.4%). Figure 2 graphically depicts between-facility variability in MOVE! utilization, rank-ordered from smallest to largest on the *x*-axis (size of the points represent facility size). Among patients under 70 (*n* = 1,468,911), who are specifically targeted for MOVE!® services, 6% (*n* = 83,140) of all veterans in the final analytic sample had at least one MOVE!® visit, with an average of 4.8 (*SD* = 8.2, median = 2.0) MOVE!® visits among those with at least one visit.

### Unadjusted predictors of MOVE!® utilization

Obese VHA patients were more likely to use MOVE!® one or more times in FY2010 if they had a higher BMI, were younger, female, not married, of minority status, had home instability, or lived in urban areas (Table 2). In addition, MOVE!® utilization was positively associated with outpatient copayment status (payment required vs. exempt), obesity diagnosis, obesity or psychiatric-related comorbidities, and obeseogenic psychiatric drug prescriptions.

**Table 2:** Patient- and facility-level correlates of MOVE! utilization among obese patients treated in the veterans health administration in fiscal year 2010

**Patient-level correlates**		
**Total unique patients**	**No MOVE! use**	**1+ MOVE! visits**
(*N* = 2,054,367)	1,964,129 (95.61%)	90,238 (4.39%)
**Age** (ref.: <40 years old)		
40–49***	9.83%	14.15%
50–59***	19.71%	28.28%
60–69***	34.40%	41.51%
70–79***	19.03%	6.54%
80 + ***	9.38%	1.10%
**BMI** (ref.: Overwt [<30 BMI])		
Class I (30–34 BMI)***	44.43%	35.62%
Class II (35–39 BMI)***	17.14%	26.93%
Class III (40+ BMI)***	8.11%	22.98%
**Gender**		
Female***	5.86%	11.91%
**Marital status**		
Married***	60.14%	52.34%
**Minority status**	298,495 total missing	
Minority***	24.93%	32.58%
**Home instability**		
Yes***	2.12%	5.76%
**Geographic location** (ref.: Highly Rural)	12,935 total missing	
Rural	11.43%	8.39%
Urban**	88.30%	91.27%
**Outpatient copay**		
Yes***	0.18%	0.23%
**Obesity comorbidity**		
Yes*	88.55%	90.44%
**Psychiatric Dx**		
Yes***	20.83%	36.56%
**Obesity–related care**		
Yes***	16.43%	87.03%
**Facility-level correlates**		
**Total facility rates (percentage)**	**Patient’s with No MOVE! use**	**Patient’s with 1+ MOVE! visits**
**Male gender** [mean (sd)]	93.84 (01.90)	93.87 (01.80)
Minority status*** [mean (sd)]	25.22 (18.41)	24.26 (16.86)
Home instability*** [mean (sd)]	02.26 (01.56)	02.51 (01.80)
**Outpatient copay** [mean (sd)]	00.18 (0.09)	00.17 (00.09)
Rural location* [mean (sd)]	11.30 (21.16)	09.66 (18.86)
**BMI** [mean (sd)]	32.78 (0.47)	32.80 (0.39)
Obesity comorbidity** [mean (sd)]	88.65 (02.67)	88.32 (02.57)
**Psychiatric Dx** [mean (sd)]	21.52 (04.07)	21.44 (03.93)
Obesogenic drugs *** [mean (sd)]	17.93 (03.10)	17.50 (02.77)
Obesity comorbidity** [mean (sd)]	88.65 (02.67)	88.32 (02.57)

Note: *p < .05, **p < .01, ***p < .001 in single-predictor, mixed-effects regression models with a random effect for VA facility. For categorical variables, significant differences are between the indicated group and the reference group.

Obese VHA patients treated at facilities with higher proportions of minority patients, home instability, and patients from urban residence were more likely to utilize MOVE!® (Table 2). In addition, facilities with a larger proportion of patients with lower rates of obesity-related comorbidities and obesogenic psychiatric medication prescriptions had higher MOVE!® utilization. Facility-level variables that were not related to MOVE!® utilization included the proportion of men, outpatient copayment required, psychiatric diagnoses, and average facility BMI.

### Adjusted predictors of MOVE!® utilization

In adjusted analyses (Table 3), veterans were more likely to use MOVE!® if they had Class III BMI (morbidly obese) (OR = 4.54 , 95% confidence interval (CI): 4.43, 4.56), were between 40–69 years old, compared to <40 (OR = 1.21-1.25), female (OR = 1.66, 95% CI: 1.62, 1.7), not married (OR = 0.94, 95% CI: 0.93, 0.95), minority status (OR = 1.36, 95% CI: 134, 1.39), home instability (OR = 1.63, 95% CI: 1.57, 1.68), living in urban areas (OR = 1.17, 95% CI: 1.15, 1.19), required to pay outpatient copayments (OR = 1.63, 95% CI: 1.57, 1.68), obesity and psychiatric-related comorbidities (OR = 1.68, 95% CI: 1.64, 1.73 and OR = 1.58, 95% CI: 1.55, 1.60, respectively), and obeseogenic psychiatric drug prescriptions (OR = 1.17, 95% CI: 1.15, 1.19). Veterans were less likely to use MOVE!® if they were seen in VA medical centers with higher rates of home instability (OR = 1.09 95% CI: 1.02, 1.16) and lower rates of obesogenic psychiatric drug prescriptions (OR = 0.92, 95% CI: 0.90, 0.95). The individual-level fixed effects (predictors) accounted for 20% of the variance in MOVE! utilization (marginal psuedo-R^2^). The facility-level fixed-effects (predictors) accounted for 2% of the variance in MOVE! utilization (marginal psuedo-R^2^). An additional 8% of the variance in MOVE! utilization was explained by between-facility variability (conditional psuedo-R^2^).

**Table 3:** **Final model of MOVE! utilization among obese patients treated in the veterans health administration (FY 2010) **^**a**^**(N=1,742,937)**

	**OR [95% CI]**	**P**
**Age** (ref.: <40 years old)		
40–49	1.21 [ 1.17, 1.25 ]	<.001***
50–59	1.25 [ 1.21 , 1.28 ]	<.001***
60–69	1.24 [ 1.20 , 1.28 ]	<.001***
70–79	0.45 [ 0.44 , 0.47 ]	<.001***
80+	0.18 [ 0.17 , 0.20 ]	<.001***
**Female gender**	1.66 [ 1.62 , 1.70 ]	<.001***
**Married**	0.94 [ 0.93 , 0.95]	<.001***
**Minority racial status**	1.36 [ 1.34 , 1.39 ]	<.001***
**BMI** (ref.: Overwt [<30 BMI])		
Class I (30–34 BMI)	1.47 [ 1.44, 1.51]	<.001***
Class II (35–39 BMI)	2.67 [ 2.61 , 2.73 ]	<.001***
Class III (40+ BMI)	4.54 [ 4.43 , 4.56 ]	<.001***
**Home instability**	1.63 [ 1.57 , 1.68 ]	<.001***
**Outpatient copay**	1.57 [ 1.35 , 1.83]	<.001***
**Geographic location** (reference: Highly Rural)		<.001***
Rural	0.95 [ 0.82 , 1.09 ]	.46
Urban	1.17 [ 1.15 , 1.19]	.02*
**Obesity comorbidity**	1.68 [ 1.64 , 1.73 ]	<.001***
**Obesogenic psychiatric drug**	1.17 [ 1.15 , 1.19 ]	<.001***
**Psychiatric diagnosis**	1.58 [ 1.55 , 1.60 ]	<.001***
**% Home instability**	1.09 [ 1.02 , 1.16 ]	<.001***
**% Obesogenic drugs**	0.92 [ 0.90 , 0.95 ]	<.001***
**Intercept**	0.03 [ 0.02, 0.06 ]	<.001***

Note: ^a^Results from mixed-effects logistic regression model with a random effect for facility. *p < .05, ***p < .001.

## Discussion

Health care systems initiating major behavioral health programs often face challenges with variable implementation and uneven patient engagement [16,17]. Identifying factors that predict both patient engagement and facility variability in utilization can help focus subsequent quality improvement efforts. This study examined patient and facility characteristics associated with MOVE!® utilization among obese VHA outpatients in 2010. Among the almost 2.5 million VHA patients meeting criteria for obesity from 140 VA facilities in 2010, 4.4% had at least one MOVE!® visit. Among patients younger than 70 to whom MOVE!® is targeted, 6% had at least one MOVE!® visit. Of note is that Office of Quality and Performance (OQP) chart review process found that 10% of "at-risk" Veterans had utilized MOVE!® in 2010 [8], which is a considerably higher rate than was found here. The chart review procedure used by OQP typically involves taking a relatively small sample of patient records and thoroughly examining clinic notes related to inpatient or outpatient visits. While chart review may be more sensitive than administrative data for identifying MOVE!® visits, the estimates from small sample chart review can be highly variable. In contrast, although administrative data utilized in this study may miss some MOVE! visits, the estimates of use have much tighter confidence intervals due to the very large sample size.

Perhaps more interesting and important than the overall utilization rate is the large variation in use across VHA facilities (ranging .05% to 16%). These data show that some facilities manage to provide MOVE!® services to nearly 4 times the national average in terms of proportion of patients served. In addition, this finding suggests that many facilities could dramatically increase the proportion of obese patients receiving these services. More research is needed to examine the barriers and facilitators of MOVE!® utilization, particularly in facilities with unusually high and low reach. Also, increasing the number of visit per patient is an important quality improvement goal since prior work has shown that having two or more MOVE!® visits is associated with at least a 5% weight loss [8].

### Patient characteristics

It is certainly promising that veterans with higher BMI, obesity-related comorbidities, and obesogenic drug prescriptions had greater MOVE!® utilization because this suggests that veterans most in need of MOVE!® obesity management services were accessing MOVE!® at higher, albeit still suboptimal, rates. Unexpected patient-level predictors of MOVE!® utilization included home instability and having a required outpatient copayment. Greater MOVE!® utilization among patients with home instability may be encouraged by their VA providers to use MOVE!® outpatient services as a part of their treatment plans.

The positive association with MOVE!® use and required outpatient copayment may be due to the fact that MOVE!® services are offered to all Veterans at no cost due to copay exemption or waiver of copays in June 2008 [18]. Having a copayment requirement may be a proxy for higher socioeconomic status and less illness severity because VA requires veterans to pay copayments if they have limited military-service related disability or higher incomes.

Patients between 40–69 years-old were more likely to utilize MOVE!® services. After age 69, there is a substantial decline in MOVE!® utilization, which may be due to the target of screening for referral (i.e., automated clinic reminders) to MOVE!® services are those obese patient younger than 70 for whom weight management are clear. A sensitivity analysis that restricted the sample to patients younger than 70 found a MOVE!® utilization rate of 6% (versus 4.4% for the entire sample of obese patients), but the coefficients for the other predictors remained nearly unchanged.

The positive association between MOVE!® utilization and urban geographic location is consistent with previous obesity management literature [8] and health services utilization (generally) [19]. Veterans living further away from the VHA hospital are less likely to attend the program, due to proximity-related barriers.

### Facility-level predictors

After accounting for several patient-level predictors in a multi-predictor mixed effects regression, some specific facility factors related to treatment context or casemix were associated with receipt of at least one MOVE!® visit. Veterans at facilities with higher proportion of patients with home instability and lower obesogenic drug prescriptions rates were more likely to access MOVE!® Regarding the positive relationship between facility-level proportion of home instability and MOVE!® use, perhaps (and similar to the individual-level relationship) there is greater facility-wide emphasis on having providers encourage their obese patients to use MOVE!® outpatient services as a part of their treatment plans. However, although statistically significant, these findings may be less clinically relevant. That is, the overall differences in MOVE! utilization by facility-level characteristics were small (accounted for 2% of variability in MOVE! use) but the sample size was large enough to detect statistical differences.

### Limitations

This study has several limitations, including a cross-sectional observational study design that was limited to patients’ treated in the VHA in FY2010, which limits causal interpretation of findings and may not generalize to earlier or later years. Another limitation is that the number of MOVE!® visits was not examined, which may result in very different findings from that of utilization based on at least 1 MOVE!® visit. Finally, there were several unmeasured variables, such as differential resource allocation across facilities, that may be associated with MOVE!® utilization that could bias these results.

Despite these limitations, this study had several strengths. Over 2 million patients treated across 140 VHA hospitals nationwide in 2010 were included in this analysis. To our knowledge, this is the largest obesity management study conducted to date. Substantial variation in MOVE!® utilization across facilities was found and several patient- and facility-level factors were associated with this variation. Although MOVE!® utilization is modest for obese Veterans, those who are in most need of this obesity management service (i.e., patients’ diagnosed with obesity and obesity-related comorbidities) are more likely to access them (albeit at a low level). However, there may still many Veterans in need who are not accessing these services. The findings from this study suggest that reducing barriers to MOVE! utilization for these patients is an important next step in disseminating MOVE!® further.

## Conclusion

The results from this study suggest that veterans most in need of obesity management services are more likely to access MOVE!® (albeit at low levels). However, substantial variation exists across VHA facilities in utilization rates and much of the variability in utilization remains unexplained. Tailored outreach and patient-education materials might target groups under-served by MOVE!®, including rural veterans and less obese veterans. In addition, determining ways to increase access and utilization of MOVE!® is an obvious implementation challenge. Provision of tele-based or web-based MOVE!® could improve access, and VA is currently developing these modalities. Further research examining lower rates of MOVE!® utilization among men and those patients’ living in rural communities is needed to guide development of interventions to improve MOVE!® receipt. Although our focus was to predict overall MOVE!® utilization, another related area we did not address was predictors of MOVE!® utilization conditional on having received other obesity care. The predictors of a model including that term answers a different question: What are the predictors of MOVE!® above and beyond the variance explained by receipt of other obesity care in general? Given the high overlap of MOVE!® and other obesity care, this latter question would be better addressed by restricting the analysis to patients who receive some obesity care of any kind. Although the focus of this study was on VHA, these results may apply to other health care systems that are initiating similar programs.

## Competing interests

AC Del Re has no financial disclosures or competing interests.

Matthew L. Maciejewski has no financial disclosures or competing interests.

Alex HS Harris has no financial disclosures or competing interests.

## Authors’ contributions

AD conceived of the study, participated in its design, performed the statistical analysis and helped to draft the manuscript. MM participated in its coordination and helped to draft the manuscript. AH participated in the design of the study and coordination and helped to draft the manuscript. All authors read and approved the final manuscript.

## Pre-publication history

The pre-publication history for this paper can be accessed here:

http://www.biomedcentral.com/1472-6963/13/511/prepub
